# Do patients with cervical dystonia present a greater risk and more fear of falling?

**DOI:** 10.1055/s-0045-1806822

**Published:** 2025-04-27

**Authors:** Sibele Yoko Mattozo Takeda, Ana Félix de Souza, Iara Ferreira Penteado, Djanira Aparecida da Luz Veronez, Hélio Afonso Ghizoni Teive

**Affiliations:** 1 Universidade Federal do Paraná, Setor de Ciências Biológicas, Departamento de Prevenção e Reabilitação em Fisioterapia, Curitiba PR, Brazil.; 2 Universidade Federal do Paraná, Setor de Ciências Biológicas, Departamento de Anatomia, Curitiba PR, Brazil.; 3 Universidade Federal do Paraná, Setor de Ciências da Saúde, Departamento de Clínica Médica, Curitiba PR, Brazil.

**Keywords:** Dystonia, Postural Balance, Gait, Rehabilitation

## Abstract

**Background** Cervical dystonia (CD) is a movement disorder characterized by involuntary contractions that affect the muscles of the cervical region, causing movements in a twisting pattern. Its chronic-progressive nature can lead to pain, impaired mobility, and greater propensity to falls.

**Objective** To verify gait and balance characteristics and their correlation with the risk and fear of falling in 46 CD patients.

**Methods** The present was a descriptive and cross-sectional study. For the assessments, we sued the Berg Balance Scale (BBE), the Tinetti Scale (also known as the Performance-Oriented Mobility Assessment, POMA), the Dynamic Gait Index (DGI), the Fall Efficacy Scale-International (FES-I), and the Functional Reach Test (FRT). The patients were recruited during their routine consultations, before the administration of botulinum toxin. For data analysis, we used the BioEstat 5.0 software (free) to verify the distribution of samples (Shapiro-Wilk test) and the correlations among variables (Spearman test) considering *p* ≤ 0.05%.

**Results** The results showed that the patients presented gait changes related to static and dynamic components of balance. Therefore, we observed a greater propensity to falls, with a correlation involving lack of balance, a greater risk of falling and, consequently, more fear of falling.

**Conclusion** We verified that changes in the balance of CD patients have a negative impact on gait, increase the risk and the fear of falling, and can lead the individual to restrict their activity levels.

## INTRODUCTION

Cervical dystonia (CD) is a movement disorder characterized by sustained or intermittent involuntary muscle contractions that trigger abnormal movements and/or postures that are generally repetitive. These dystonic movements have a torsion pattern, and normally present with dystonic, action, or postural tremor, which can be initiated or exacerbated by voluntary movements. 1 2 3 4

The muscles in the cervical region (specifically the muscle spindles) have sensory receptors that carry information related to the body's positioning in space to the higher motor control centers. Therefore, under normal conditions, vibratory stimuli in the cervical region can influence the positioning of the body in space. But when the same vibratory stimulus is provided to CD patients, this interference does not seem to occur. It is assumed that, due to the incorrect positioning of the head caused by the disease, the motor control centers disregard the sensory information coming from the cervical muscles. Thus, “wrong” feedback is prevented from being processed and, consequently, incorrect postural adjustment and its consequences are avoided. 5 6

Balance plays a key role in maintaining posture; as such, it can be defined as the ability to maintain the positioning of a body that oscillates minimally (static balance), or as maintaining posture during the performance of a certain motor skill which disturbs the body's orientation (dynamic balance). 7 Thus, a lack of balance negatively affects function, leading to disability, which commonly restricts activity levels, triggering abnormal compensatory motor behaviors and requiring aid from devices or other types of assistance. Severe imbalance can lead to falls and may cause secondary injuries. 6

Lesions to the nervous system lead to significant changes in gait, and the method of locomotion may indicate a specific central area of injury to the nervous system, in addition to psychiatric conditions or lesions to the vestibular system. 8 Human gait is a complex spatiotemporal system that involves the structures and functions of the musculoskeletal system. Although gait is a characteristic with high individuality, there are degrees of similarity among individuals which enable the establishment of a typical gait pattern. However, under pathological conditions of neuromuscular etiology, the efficiency of gait may be reduced due to the influence of different factors. 9 Under this situation, postural instability and balance disorders can lead to a fear of falling, a restriction of activities and locomotion, physical deconditioning, and changes in gait patterns. 10

Considering the clinical signs of CD, as well as the importance of stability and alignment of the head and trunk to ensure effective postural adjustment, balance, and, consequently gait, may be affected by this condition, which may increase the propensity to falls, as well as the fear related to them. In this context, the objective of the present study was to evaluate gait and balance and their correlation with the risk and fear of falling in CD patients monitored by a reference service at a Brazilian public hospital.

## METHODS

The present was a descriptive, cross-sectional and propective study of a qualitative and quantitative nature, 11 which was approved by the Ethics in Research Committee of the Health Sciences Sector of Universidade Federal do Paraná, in the city Curitiba, Brazil, under number 1516871. For the development of the current study we considered the Strengthening the Reporting of Observational Studies in Epidemiology – (STROBE) statement.

**Figure 1:**
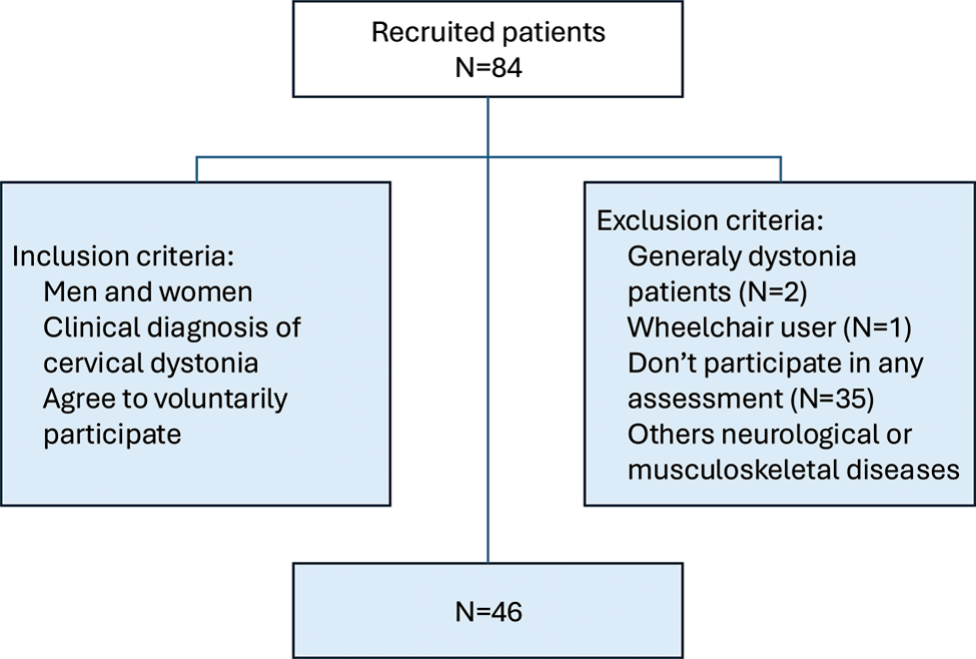
Participant selection and research procedures.

The sample was non-probabilistic, 12 and it consisted of subjects who met the eligibility criteria and were being monitored by a specialized reference service. Among them were men and women aged between 33 and 65 years, with a clinical diagnosis of CD dystonia, who voluntarily agreed to participate of the study. The exclusion criteria were patients with generalized dystonia, those using a wheelchair, subjects who failed complete any of the assessments, and patients presenting other neurological and/or musculoskeletal diseases ( Figure 1 ). The participants were recruited through an invitation made during return consultations. The initial patient assessment consisted of collecting data related to the direction of the dystonic movement, the onset of the disease, and the presence of pain. All assessments were performed once, before the administration of botulinum toxin by the responsible doctor.

### Balance assessment

Balance was assessed through the Berg balance scale (BBS), 10 13 which contains 14 items that assess an individual's ability to maintain balance while sitting, standing, standing on one leg, maintaining a tandem posture, decreasing the base of support, crossing steps, reaching, turning around, and looking over their shoulders. Each test is scored on a five-point scale ranging from 0 (unable to perform the task) to 4 (performs the test independently), based on the evaluation of the time it took and the method of executing the movement. 14 As such, the sum of all the evaluated items varies from 0 to 56 points, and a higher score indicates better the functional balance.

The BBS score has been shown 14 15 to present significant usefulness in predicting the risk of falling. A score ≤ 36 points indicates a risk of falling of 100%, a score ≤ 45 points indicates poor balance, leading to an increased risk of falling, and it has been indicated as a predictor of falls if associated with a history of imbalance.

The functional reach test (FRT) was used to evaluate anterior functional reach and to identify dynamic changes in postural control, providing quantitative information about an individual's ability to move anteriorly with their feet fixed to the ground. This test was performed with the individual standing, positioned laterally to the wall, with their shoulders positioned at a distance of 15 cm and flexed at 90°, with the upper limbs extended. The initial measurement was performed in the position in which the styloid process of the ulna was parallel to the measure tape fixed to the wall. 7 Under these conditions, the displacement distance was measured using a measuring tape, and the final result was calculated as the average of the displacement measurements in three attempts. 16

### Tinetti Scale

The Tinetti Scale, also known as the Performance-Oriented Mobility Assessment (POMA) evaluates balance, gait, and the risk of falling. It consists of a 16-item test, including 9 that assess balance, and 7, gait. Gait speed, distance, step height, step symmetry, trunk oscillation, and the ability to turn and perform movements with the eyes closed were evaluated. The scores, which range from 0 to 1 or 0 to 2 depending on the item, on each movement were added. The total score was calculated as the sum of the balance (maximum: 16 points) and gait (maximum: 12 points) domains, yielding a potential maximum of 28 points. The lower the total value, the lower the physical ability and, consequently, the greater the risk of falling. 17 18

### Dynamic Gait Index

The Dynamic Gait Index (DGI) was used to assess functional mobility. This score comprises eight activities performed on a flat surface at a pre-established distance of 6 m. Scores were classified as normal ( *n* = 3), mild ( *n* = 2), moderate ( *n* = 1), or severe ( *n* = 0). A total score result of less than or equal to 19 points corresponds to a significant change in functional mobility, and has previously been validated as a predictor of the risk of falls. 19

### Falls Efficacy Scale-International

The Falls Efficacy Scale-International (FES-I) is used to assess the fear of falling associated with a certain frequency of falls. The questionnaire comprises 16 items related to activities of daily living such as cooking; bathing; changing clothes; going shopping; attending social events; walking around the neighborhood; and walking on uneven surfaces, ramps, and slippery surfaces. 19

For each task in the questionnaire, the patients must mention their concerns about falling. The four available response options are as follows: 1–not at all worried; 2–slightly worried; 3–very worried; and 4–extremely worried. The total score ranges from 16 (absence of concern) to 64 points (extreme concern). Furthermore, the FES-I associates the fear of falling with the frequency of falls. As such, the closer the score is to 64, the greater the fear of falling. Likewise, a score ≥ 23 indicates a fear of falling associated with sporadic falls, while scores ≥ 31 indicate a fear of falling associated with recurrent falls. 19

The choice of instruments to assess balance, the risk of falling, the fear of falling, and gait was based on the fact that these issues have been evaluated from different perspectives. Furthermore, they are inexpensive and easy to apply. 19

To analyze the data obtained, the distribution of the sample was verified using the Shapiro-Wilk test. The Spearman's correlation test was used to correlate the data, with *p* < 0.05 indicating statistical significance. The BioEstat5.0 software (free) was used for the statistical analysis. The magnitude scale proposed by Hopkins was used to interpret the correlation coefficients, with the following definitions of scores: < 0.1–trivial; 0.1 to 0.29–small; 0.30 to 0.49–moderate; 0.50 to 0.69–high; 0.70 to 0.90–very high; and > 0.90–almost perfect.

## RESULTS

### Characterization of the participants

A total of 46 CD patient were included ( Figure 1 ): 39.13% were men ( *n* = 18) with a mean age of 49 ± 16 years, and 60.87% were women ( *n* = 28) with a mean age of 53 ± 13 years.

Complaints of pain in the cervical region were reported by 45.65% ( *n* = 21) of the sample. Another important sign of CD was tremor, with head tremor found in 69.56% ( *n* = 32) of the patients, head and upper limb tremors, in 4.34% ( *n* = 2), and tremor only in the upper limbs in 2.17% ( *n* = 1) of the subjects. However, 23.91% ( *n* = 11) of the patients did not report tremors.

Regarding the direction of the cervical movement, 2.17% ( *n* = 1) of the patients developed an anterocollis-type pattern, while 71.74% (n = 33) developed laterocollis, 8.69% (n = 4), retrocollis, and 15.22% (n = 7), torticollis. Only 2.18% (n = 1) did not show any clear pattern of movement direction. Therefore, in the present study, laterocollis was the most prevalent direction of movement, followed by torticollis.

Regarding the “diagnosis time” means the age at diagnosis was of 13.94 ± 10.26 years for men, and of 13.86 ± 9.52 years for women, with men undergoing therapeutic follow-up for 10.26 ± 6.58 years, and women, for 9.43 ± 7.97 years.

### Berg Balance Scale

When analyzing balance using the BBS, 20 we could verify important changes in balance to maintain different postures. Approximately 17.39% ( *n* = 8) of the patients obtained scores ≤ 36, which predict a 100% risk of falling. Meanwhile, 28.26% ( *n* = 13) achieved a score ≤ 45, indicating an increased risk of falling, and 54.34% ( *n* = 25) had a predisposition to a low risk of falling, with scores > 45 points. Therefore, all patients presented with balance deficits and some propensity to fall ( Table 1 ).

**Table 1:** Risk of falling according to different scales

	Men: n (%); score	Women: n (%); score	Results
**Dynamic Gait Index**	11 (61.1%); < 19	17 (60.71%); < 19	Risk of falling
**Falls Efficacy Scale-International-Brasil**	10 (55.56%); ≥ 31	16 (57.14%); ≥ 31	Recurring falls
**Berg Balance Scale**	3 (16.67%); ≤ 36	5 (17.85%); ≤ 36	100% risk of falling
**Tinetti Scale (Performance-Oriented Mobility Assessment, POMA)**	4 (22.22%); < 19	5 (17.85%); < 19	High risk of falling
**Functional Range Test**	3 (16.67%) < 15 cm	8 (28.57%); < 15 cm	Fragility and risk of falling

### Functional Range Test

The FRT, which assesses static balance, plays an important role in the assessment of activities of daily living, in addition to signaling fragility and the risk of falling. In the present cohort, 23.91% ( *n* = 11) of the patients had a score < 15 cm, indicating fragility and risk of falling. 16 In addition, 17.39% of the subjects had a BBS score < 45, indicating significant impairment in balance and a very high risk of falling ( Table 1 ). By correlating the BBS and FRT ( *p* < 0.0001; r = 0.73), we verified the relationship between the static and dynamic components of balance.

### Tinetti Scale and the Dynamic Gait Index

Regarding gait, which was evaluated through the POMA, 19.56% of the patients scored < 19 points, indicating poor physical ability to walk and, consequently, a high risk of falling. In addition, 34.78% ( *n* = 16) presented a moderate risk of falling (scores ranging from 19–24 points), while 45.65% ( *n* = 21) had a score > 24 points, indicating no risk of falls. Similarly, in the DGI, 60.87% ( *n* = 28) of the participants achieved scores , 19, indicating reduced walking ability, which may result in a greater risk of falling.

### Fear of falling (FES-I-BRASIL)

Regarding the fear of falling (assessed using the Brazilian version of the FES-I, the FES-I-BRASIL), the following results were obtained: 17.39% ( *n* = 8) achieved scores ≥ 23, indicating the fear of falling associated with sporadic falls. In addition, 56.52% ( *n* = 26) presented scores ≥ 31, which associate the fear of falling with recurrent falls. 21

## DISCUSSION

The data obtained confirm the higher prevalence of the disease in females, which corroborates previous findings, such as those of a study 22 with 135 patients with dystonia in which 69.9% were women. Likewise, clinical studies 23 on CD have found a female predominance among patients, with a female:male ratio of 1.5:1. 23 Although women are more affected, Ledoux et al. 24 reported no differences in the degree of commitment between men and women. Regarding age, CD is more prevalent in adulthood, as highlighted by Queiroz et al. . 3 Within this context in a study with more than 1 thousand patients with CD, Comella and Bathia 25 found a mean age of 53.2 ± 11.9 years.

Pain has been previously identified as one of the main complaints of CD patients, reported by 50% of the subjects in certain studies. 25 26 These findings are similar to those observed in this study, in which 45.65% of the participants reported pain complaints.

Overall, in the cohort of the present study, head tremors were more common than tremors in other regions of the body. The same trend was observed in a study 27 using the medical records of 185 CD patients from the Extrapyramidal Diseases Research Sector of Escola Paulista de Medicina, Universidade Federal de São Paulo, in which some form of tremor was observed in 33% of the cases. The authors 27 also mentioned another study, 29 with a large number of subjects, in which tremors occurred in 71% of the CD patients, 60% whom reported head tremors.

Considering the characteristic movements of CD, Jiménez-Jiménez et al. 28 reported that torticollis, laterocollis, anterocollis, and retrocollis are the most common directions respectively, indicating the involvement of important muscle groups in the maintenance of head and trunk stability. The sternocleidomastoid, trapezius, splenius capitis, scalene platysma, and levator scapulae are the primary muscles involved in CD. 29 30 Therefore, the data demonstrated in these studies indicate that laterocollis and torticollis are the most common forms.

Since the time until diagnosis and the duration of the treatment depend on the characteristics of CD, periodic monitoring by a multidisciplinary team is necessary to minimize its repercussions.

### Berg Balance Scale

Because of the chronic nature of CD, as well as the fact that it most frequently affects adults and the elderly, it is assumed that static and dynamic balance are compromised, with the main functional impact on gait.

Similarly, Barbosa and Warner 31 observed that CD patients present with proprioceptive impairment, which leads to disturbances in dynamic balance. Furthermore, head position, along with abnormalities in the vestibular and visual systems, contribute to this instability in dynamic control. Overall, 54.34% of the patients evaluated, of either gender, had balance changes and, consequently, a predisposition to falls.

### Functional Range Test

In a similar study, De Castro et al. 19 reported difficulty in modifying the gait pattern given the activities required by the scale.

By correlating the BBS and the FRT ( *p* < 0.0001; r = 0.73), we verified the relationship between the static and dynamic components of balance, with the combination of these factors being essential for the stability of the body at rest and during movement. Barr et al. 32 reported that CD patients present a deficiency in postural control, in addition to a loss of mobility when turning, walking or transferring positions. This has a negative impact on balance, slowing down balance reactions and reducing walking speed. Barr et al. 32 further showed that the patients report frequent fear when performing physical activity. This correlation was further verified by Mittal et al., 33 who showed that women presented with smaller anterior displacements when compared with men, which is in line with the findings of the current study. Overall, research suggests that factors such as gender, age and height can influence these values.

The patients in the present study, as well as those in the study by Barr et al., 32 showed a reduction in reaction time for decision and response, as found in ∼ 10.71% of the patients evaluated who did not obtain a score in the functional range. This was because they were unable to project their bodies forward, reporting fear of falling. Furthermore, the position of the neck leads to a reduction in visual reliability and balance, impacting proprioception and, consequently, gait functionality and balance. 32

### Tinetti Scale and the Dynamic Gait Index

The gait pattern in patients with neuromuscular deficits, when affected by synergistic organization and nonintegrated primitive reflexes, is characterized by a decrease in straightening and balance reactions. Furthermore, body dissociation and coordination are influenced, causing anterior or posterior tilts. 18

In the present study, based on the evaluation of the POMA, most patients did not presented risk of falling. Conversely, previous studies, such as that by Barr et al., 32 showed that these patients had a greater functional limitation in walking, which led them to walk at a slower speed and with a longer contact time with bipedal support on the ground. However, most patients achieved higher physical ability scores.

The clinical signs of CD indicate that balance and gait are affected. Normal gait is impaired by pathological states, particularly neuromuscular conditions that involve weakness, contractures, and pain. 19 When correlating the BBS and DGI, a positive and strong relationship was found between the scales ( *p* < 0.0001; r = 0.68), indicating that the higher the BBS score, the better the balance and lower the risk of falling in these patients, with 54.34% ( *n* = 25) predicted as presenting no risk of falling and 60.86% ( *n* = 28), worse walking ability.

### Fear of falling (FES-I-BRASIL)

Thus, the aforementioned values portray the relationship involving the lack of static and dynamic balance, the risk of falling, and the fear of falling. Furthermore, when the components of balance, gait, and fear of falling were correlated, we observed that the more affected the static and dynamic balance, the more compromised the gait and the greater the risk of falling, and, consequently, the greater the fear of falling ( *p* < 0.05).

In the aging process, body balance declines; therefore, and due to the scarcity of studies related to the fear of falling in CD patients, we performed a comparison of the results of the current research with those of a previous Brazilian study with 53 elderly people aged > 60 years. 34 In which the score obtained in the evaluation using the FES-I-BRASIL was of 26.5 ± 7.3.

Corroborating the findings of the present study, Mazo et al. 35 and Lopes et al. 36 observed that 20 to 60% of the subjects experienced a fear of falling, even if no falls had occurred. Therefore, the findings of the current study reinforce the idea that the preexistence of balance disorders can increase the risk and fear of falling.

Overall, the results of the present study show that CD is associated with impaired static and dynamic balance. This has a negative impact on gait performance, increasing the risk and fear of falling. However, due to the scarcity of literature on the physical and functional impact of cervical dystonia on the lives of these patients, future studies with larger samples and randomized methodological approaches are needed to develop effective therapeutic strategies.
